# Effect of different periodontal ligament simulating materials on the incidence of dentinal cracks during root canal preparation

**DOI:** 10.15171/joddd.2018.030

**Published:** 2018-09-18

**Authors:** Ajita Rathi, Prateeksha Chowdhry, Mamta Kaushik, Pallavi Reddy, Roshni, Neha Mehra

**Affiliations:** Department of Conservative Dentistry and Endodontics, Army College of Dental Sciences, Secunderabad, Telangana, India

**Keywords:** Periodontal ligament simulation, dentinal defects, stereomicroscope

## Abstract

***Background.*** The present study was undertaken to evaluate the incidence of dentinal cracks during root canal preparation with different periodontal ligament simulating materials in vitro.

***Methods.*** Seventy freshly extracted human mandibular first premolars were selected and divided into 7 groups in terms of simulating material: group 1: polyether impression material; group 2: polyvinyl acetate adhesive; group 3: polyvinyl siloxane impression material; group 4: cyanoacrylate adhesive; group 5: epoxy resin adhesive; group 6: positive control, without any periodontal ligament simulation; and group 7: negative control, where neither a periodontal ligament simulating material was used nor canal preparation was carried out. Root canal preparation was carried out in all the groups followed by sectioning of roots at 3 mm, 6 mm and 9 mm. The sections were evaluated under a stereomicroscope at ×2.5 for the presence or absence of cracks. Chi-squared test was used to compare the appearance of defective roots between the different experimental groups.

***Results.*** The least number of cracks were found in the negative control group, followed by group 1 where polyether impression material was used for periodontal ligament simulation. The difference was significant with a P-value of 0.002 for coronal sections.

***Conclusion.*** Under the limitation of the present study, polyether and polyvinyl siloxane (light body) can both be used for simulation of periodontal ligament.

## Introduction

The success of endodontic treatment depends primarily on thorough chemomechanical debridement. ^1^ A long series of instruments from stainless steel hand files to several rotary drills to nickel titanium files for shaping canals have been introduced. The NiTi rotary instrumentation offers advantages such as the maintenance of original shape and curvature of the canal, reduced likelihood of procedural errors, shortened treatment time and an ideal tapering canal form for obturation.^2^

During biomechanical preparation, shaping of canal takes place by the contact between instruments and dentin walls, creating many momentary stress concentrations in dentin.^3^ These stress concentrations have the potential to induce dentinal defects and microcracks or craze lines. Since, the applied stresses caused by root canal obturation, retreatment and re-peated occlusal forces get exponentially amplified at the tip of the pre-existing defects, these dentinal de-fects, in turn, are associated with increased VRF (ver-tical root fracture) susceptibility.^4,5^

Whenever occlusal forces are applied onto the tooth, the load gets distributed through the bone via the periodontal ligament, thereby preventing the fracture of the tooth. The periodontal ligament is a soft connective tissue which joins the tooth root to the alveolus and provides anchorage to the tooth in the alveolar bone. Its thickness ranges from 0.1 to 0.3 mm. It helps in absorbing occlusal loads and distributes them towards the bone, thereby preventing fracture of the teeth.^6^

The periodontal ligament, if possible should be reproduced in the laboratorial experiment in order to simulate the clinical reality more accurately.

Considering that during biomechanical preparation stresses are induced, leading to dentinal defects and micro-cracks and craze lines, periodontal ligament simulating materials may also interfere in stress distribution.^2,3^ Therefore, the aim of the present study was to characterize the effect of different artificial periodontal ligament simulation techniques on the development of dentinal cracks during biomechanical preparation.

## Methods

### Sample preparation

Seventy freshly extracted human mandibular first pre-molars with straight root canals and mature apices were selected. The teeth were cleaned of calculi, soft tissues and debris with hand scalers and kept in saline solution (0.9% sodium chloride in distilled water) at 4°C for not more than one month after extraction. The external root surface was inspected under a stereomi-croscope (×2.5) to exclude any external defects or cracks. Mesiodistal and buccolingual digital radio-graphs (RVG) were taken to ensure that each tooth had similar root dimensions and also to verify the presence of a single root canal. Endo access bur #2 (Dentsply, Maillefer, Tulsa Dental Specialities) was used to prepare access cavities. The patency of the ca-nal was checked and the working length was deter-mined using a #15 K-file (Mani Inc.) until it was vis-ible at the apical foramen.

The mesiodistal width at the mid-root region of each tooth was measured using a Vernier caliper before dipping in molten wax. Twenty teeth were randomly divided into the positive and negative control group (n=20). The remaining fifty teeth were dipped in molten wax to achieve a 0.2‒0.3-mm thick wax layer all around the root, starting 1 mm below the CEJ. The thickness of the wax layer was confirmed by measuring the thickness of the root with a Vernier caliper before and after immersion (excess wax was carved off using a Lacron’s carver). All the seventy teeth were then mounted in self-curing acrylic resin up to the CEJ. The teeth were retrieved from the acrylic molds and the wax was scraped off from the root surface as well as the resin cylinder ‘sockets’. The fifty teeth were then randomly divided into five experimental groups apart from the control groups.

### Groups

Five periodontal ligament simulating materials were used to fill the space created by the molten wax and the root was immediately repositioned into their respective cylindrical ‘sockets’. Any excess material was removed. In group 1 (n=10) polyether impression material (Impregnum Soft Light Body, 3M ESPE) was used; in group 2 (n=10) polyvinyl acetate adhesive was used; in group 3 (n=10) polyvinylsiloxane impression material (Dentsply Aquasil, Light body) was used; in group 4 (n=10) cyanoacrylate adhesive was used; and in group 5 (n=10) epoxy resin adhesive was used. The remaining twenty teeth that were not dipped in molten wax were randomly divided into two control groups: group 6 (n=10) positive control, without any periodontal ligament simulation and group 7 (n=10) where neither a periodontal ligament simulating material was used nor were the canals prepared.

### Root canal preparation

All the canals were prepared using ProTaper Universal Rotary system (Dentsply, Tulsa Dental Specialties, USA) up to #F4. The canals were irrigated with 5 mL of 5% sodium hypochlorite (Vishal Dentocare, Pvt Ltd, Ahmedabad, Gujarat), 5 mL of saline and 5 mL of 17% EDTA (Dentwash, Prime Dental, Bhiwandi, India) between each instrument change, followed by a final rinse with 2 mL of distilled water. A single experienced operator performed all the procedures.

### Sectioning and stereomicroscopic observations

All the roots were cut horizontally at 3, 6 and 9 mm from the apex with a low-speed saw under water cooling. The slices were then viewed through a stereomicroscope, and images of each section were captured at ×2.5 magnification using a digital camera attached to the stereomicroscope. Each specimen was checked by 2 operators for the presence or absence of dentinal cracks.

Only sections having ‘complete cracks’ were taken into consideration (‘incomplete cracks’ and ‘craze lines’ were excluded). Any disagreement between the two examiners was resolved by discussion and a consensus was reached.

**Figure 1:**
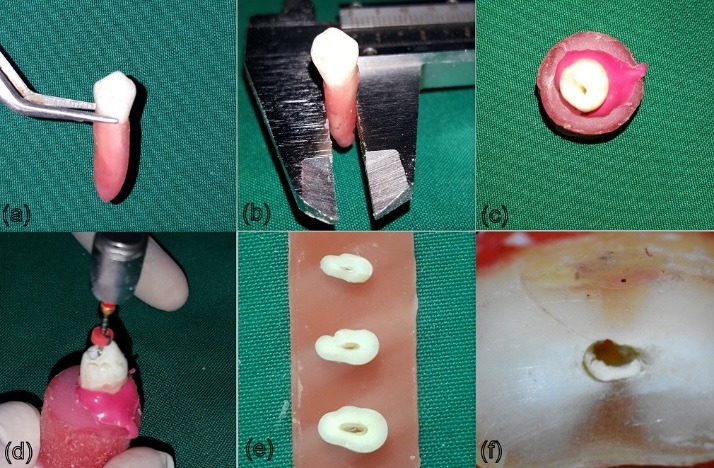
(a) teeth dipped in molten wax, (b) measure-ment using Vernier caliper, (c) excess polyvinyl silox-ane material, (d) biomechanical preparation and (e) horizontal sections at the three levels, and (f) section viewed under stereomicroscope at 2.5X showing a ‘complete crack’

The methodology is depicted in Figure 1.

### Statistical analysis

The results were expressed as the number of defected roots in each group. Chi-squared test was used to compare the appearance of defective roots between the different experimental groups.

## Results

**Figure 2:**
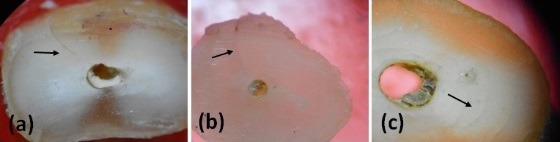
(a) ‘complete crack’, (b) incomplete crack’, and (c) ‘craze lines’.

The incidence of crack formation was highest in the positive control group (group 6), followed by perio-dontal ligament simulation with polyvinyl acetate ad-hesive (group 2), while the least number of cracks was found in the negative control group (group 7). This information is depicted in Table 1 and Figure 2. The inter-observer reliability was κ=0.85 and intra-ob-server reliability was κ=0.94 for observer A and κ=0.88 for observer B. There was a statistically sig-nificant difference between the coronal and middle and coronal and apical sections among the different experimental groups (P=0.002 for coronal sections).

**Table 1:** Incidence (number) of dentinal cracks in different groups at different levels in the root

**Sections**	**Group 1**	**Group 2**	**Group 3**	**Group 4**	**Group 5**	**Group 6**	**Group 7**
**Apical**	2	3	1	1	2	3	0
**Middle**	1	5	3	4	3	5	1
**Coronal**	0	3	1	5	3	6	1
**total**	3	11	5	10	8	14	2

## Discussion

The aim of this study was to evaluate the incidence of dentinal cracks during root canal preparation with different periodontal simulating materials in vitro. It has been reported that simulation of periodontal ligament is essential to determine stress distribution as close as possible to the clinical situation.^7,8^ The periodontal ligament is an important structure for stress distribution generated by load application over teeth. Whenever load is applied, there is compression of the periodontal ligament fibers and the tooth gets dislodged slightly. The bone gets distorted in the direction of root movement. As the tooth is forced within its alveolus, the initial low resistance of periodontal fibers against tooth displacement progressively increases.^9^ Once the periodontal fibers achieve maximum load resistance, similar to a hydraulic system, the periodontal membrane gets rigid, transferring the load to the bone support. The stress then gets distributed to the bone on all the root surfaces. Rees et al (2001) analyzed the importance of periodontal ligament through a finite element analysis, showing that it is mandatory to include the characteristics of both periodontal ligament as well as the alveolar bone.^9^

Freshly extracted mandibular premolars were selected as these teeth are probably more prone to getting influenced by forces during instrumentation due to their small dimensions and thin dentinal walls. It is unlikely for large tapered files to induce cracks in other teeth if they are not able to induce cracks in the premolars.^7^ A 0.2‒0.3-mm-thick layer of molten wax all around the root surface was used to simulate periodontal ligament, which in turn was later occupied by different simulating materials.^10^

In the present study, ProTaper Universal rotary system was used to prepare the root canals. The larger apical taper of the finishing files of this system generate increased stresses on the dentinal walls as compared to other rotary systems. This increases the incidence of dentinal cracks.^11,12^

The dentinal defects were classified as having ‘no defects’, ‘complete cracks’, ‘incomplete cracks’ and ‘craze lines’. ‘No defect’ was defined as root dentin without any lines or cracks on the external or internal surface of the root. ^‘^ Incomplete crack’ was defined as a line extending from the canal wall into the dentin without reaching the external surface; ‘complete crack’ was defined as a line extending from the root canal wall to the outer surface of the root. ‘Craze lines’ were defined as all the other lines that did not reach any surface of the root or extended from the outer surface into dentin but did not reach the canal wall.^13^ Only sections having complete cracks were counted in the study as dentinal defects because these could be caused due to forces induced during extractions.

Many other methods have been described such as stress distribution measurements, observations of the presence of defects in tooth sections and resistance of the root canal treated tooth to root fracture.^14-16^ The latter method uses application of an external force until the root fractures.^17^ The sectioning methodology used in the present study is in accordance with the methodology described where no external forces were applied on the teeth.^18^ Moreover, the effect of root canal preparation on the root canal walls and the adjacent dentin was observed directly.

The polyether impression has a non-linear and viscous behavior when submitted to external stress, which is similar to the behavior of periodontal ligament. The elastic properties of periodontal ligament were evaluated and it was found that the elastic modulus varied according to the load applied.^19^ Another study by Jamani et al evaluated the elastic modulus of Impregnum F which was found to have an elastic modulus closer to human periodontal ligament.^20^ The values for the human periodontal ligament and elastomeric impression material is given in Table 2. The elastic modulus of the other periodontal ligament simulating materials, i.e. polyvinyl acetate, cyanoacrylate and epoxy resin, is very high; hence they were not efficient in simulating the periodontal ligament as compared to the elastomeric impression materials. Based on the values observed in the present study, polyether impression material might be a good choice for periodontal ligament simulation. In summary, simulation of the periodontal ligament is more important than the material used for simulation.

**Table 2:** Young’s Moduli of human periodontal ligament (MPa)

**Range of load (N)**	**Subject number 1**	**Subject number 2**
0‒0.5	0.13±0.2	0.11±0.03
0.5‒1.0	0.26±0.08	0.23±0.05
1.0‒1.5	0.40±0.09	0.48±0.14
1.5‒2-0	0.69±0.15	0.96±0.10

Every research should be an attempt at improving the treatment of the patients. Since an in vitro experiment should represent the intraoral environment, the periodontal ligament must be simulated.

### Limitations

One important limitation of the study was that the sec-tioning methodology used does not permit evaluation of pre-existing defects, whereas micro-computed to-mography (CT) imaging is a non-destructive method and has a much higher definition than stereomicros-copy. It allows a very large number of sections per tooth to be analyzed. Ceyhanli et al., however, found that pre-instrumentation and post-instrumentation im-ages did not match perfectly as the hundreds of slices made by micro-CT are not easy to assess and some microcracks might even get overlooked.^21^

## Conclusion

Polyether and polyvinyl siloxane (light body) can both be used for simulation of periodontal ligament.

## Competing interests

The authors declare that the there are no conflicts of interest.
